# A Structural Variant in the 5' Regulatory Region of *DIAPH3* Segregates with Postlingual Hearing Loss and Auditory Neuropathy in a Multigenerational Brazilian Family

**DOI:** 10.1055/a-2832-6312

**Published:** 2026-05-05

**Authors:** Karina Lezirovitz, Danillo Alencar-Coutinho, Stella Diogo-Cavassana, Rafaella Abreu-Oberhuber, Juliana Sampaio-Silva, Maria Eduarda Paramo-Neto, Regina Célia Mingroni-Netto, Carlos Kazuo Taguchi, Ronaldo Carvalho Santos-Junior, Ana Carla Batissoco, Jeanne Oiticica, Ricardo Ferreira Bento

**Affiliations:** 1 Laboratory of Molecular, Cellular, and Translational Genetic Otolaryngology (LIM32), Hospital das Clínicas, Faculdade de Medicina, Universidade de São Paulo (HCFMUSP), São Paulo, SP, Brazil; 2 Otorhinolaryngology Department, Faculdade de Medicina, Universidade de São Paulo (FMUSP), São Paulo, SP, Brazil; 3 Department of Genetics and Evolutionary Biology, Centro de Pesquisa Sobre o Genoma Humano e Células-Tronco (CEGH-CEL), Instituto de Biociências, Universidade de São Paulo (IBUSP), São Paulo, SP, Brazil; 4 Department of Speech-Language Pathology, Universidade Federal de Sergipe, Aracaju, SE, Brazil; 5 Department of Otolaryngology, Faculdade de Medicina da Universidade Federal de Sergipe, Aracaju, SE, Brazil

**Keywords:** hearing loss, sensorineural, auditory neuropathy spectrum disorder (ANSD), complete exome sequencing, insertion-deletion mutation, *DIAPH3* gene

## Abstract

**Introduction:**

Hearing loss is genetically diverse, as it is caused by alterations in many genes and diverse inheritance patterns. While most causative variants are found in coding regions, noncoding regulatory variants can alter gene expression and may not be detected by standard exome sequencing analysis.

**Objective:**

To identify the genetic cause of autosomal dominant postlingual hearing loss in a large Brazilian family with 39 affected individuals and to describe their clinical features.

**Methods:**

Comprehensive audiological and imaging evaluations were conducted. Whole-exome sequencing on four affected subjects, supplemented by structural-variant analysis, identified a candidate variant later confirmed by quantitative polymerase chain reaction (qPCR), PCR, and Sanger sequencing.

**Results:**

Audiological testing of 61 relatives identified 31 individuals with progressive sensorineural hearing loss or auditory neuropathy linked to autosomal dominant inheritance. Genetic analysis revealed a *DIAPH3* 5'UTR deletion-insertion (c.-216_-46delinsAAGAA) that perfectly cosegregated with this specific phenotype. Notably, eight other affected relatives exhibited different clinical forms of hearing loss but did not carry the *DIAPH3* variant, suggesting separate underlying causes for their symptoms.

**Conclusion:**

Identification of this regulatory structural variant supports the role of *DIAPH3* in the etiological diagnosis of postlingual hearing loss and auditory neuropathy, with direct implications for genetic counseling and clinical management.

## Introduction

Hearing is a fundamental sensory function for communication and environmental awareness. Hearing loss (HL), whether partial or complete, has profound social, educational, and economic consequences worldwide. 1 Hearing loss is highly heterogeneous, with hundreds of genes implicated in monogenic forms involving diverse molecular mechanisms and inheritance patterns. 2 3 Among these, autosomal dominant forms frequently present as progressive postlingual HL and may exhibit considerable phenotypic variability within and across families. 4

Auditory neuropathy spectrum disorder (ANSD) represents a distinct clinical phenotype characterized by disproportionately poor speech perception relative to pure-tone thresholds. 5 It is defined electrophysiologically by the preservation of cochlear outer hair cell function, as demonstrated by intact otoacoustic emissions (OAEs), in the presence of impaired neural encoding, and reflected by abnormal or absent auditory brainstem responses (ABRs). These defects may occur at the level of inner hair cells (IHCs), ribbon synapse, or auditory nerve, and can be presynaptic, synaptic, or postsynaptic in origin. 6 7 The term *spectrum disorder* reflects the variability in age at onset (congenital, childhood, or adult) and clinical severity, ranging from mild auditory desynchrony to profound dysfunction. 5 7

Auditory neuropathy spectrum disorder can result from genetic, environmental, or multifactorial causes. 8 Congenital infections, ototoxicity, hypoxia, noise exposure, and metabolic stressors have been reported as risk factors, and ∼ 40% of cases are estimated to have a genetic basis, including syndromic and non-syndromic forms. 8 Inherited ANSD displays marked genetic heterogeneity, with autosomal recessive, autosomal dominant, and, less frequently, X-linked patterns reported. 2 9 10 11 12 Only a subset of hearing loss-associated genes causes the classic ANSD electrophysiological profile, typically those affecting the IHC-synapse-auditory nerve interface. For example, DFNB9, associated with pathogenic variants in *OTOF* , which encodes otoferlin, a calcium sensor essential for synaptic vesicle exocytosis, represents the most common autosomal recessive form and is a major target for gene therapy trials. 12 13

Variants in the diaphanous-related formin 3 *DIAPH3* gene have been associated with autosomal dominant auditory neuropathy (AUNA1). 11 14 Diaphanous-related formin 3 encodes a formin protein involved in actin nucleation and elongation, cytoskeletal dynamics, and microtubule stabilization, processes important for cell polarity, vesicle trafficking, and synaptic structure. 11 14 15 Regulatory alterations affecting its expression have been implicated in disease pathogenesis, as a non-coding 5'UTR (untranslated region) variant causing *DIAPH3* overexpression underlies AUNA1. 11 14 15

Despite advances in next-generation sequencing, establishing the molecular diagnosis of hereditary HL remains challenging. 16 17 18 Conventional exome sequencing primarily targets coding regions and may fail to detect structural or regulatory variants located in untranslated or promoter regions. Improved detection and interpretation of noncoding variants are, therefore, essential to refine molecular diagnosis, particularly in underrepresented populations. 16 17 18

The present study aimed to characterize the clinical phenotype and identify the underlying genetic etiology of autosomal dominant postlingual HL within a large group of consanguineous family members from the rural community of Pé da Serra do Bilau, Sergipe, Brazil. In this isolated population, the condition has been transmitted vertically across multiple generations, affecting males and females equally. This pattern is consistent with autosomal dominant inheritance. However, geographic isolation and reported consanguinity may have contributed to the persistence and segregation of the trait within the community.

Building upon this clinical foundation, we present the characterization of 31 family members exhibiting progressive postlingual HL and electrophysiological features consistent with auditory neuropathy. Our molecular analysis identified a novel structural variant in the 5'UTR of *DIAPH3* , which is predicted to disrupt regulatory elements and increase gene expression, aligning with the proposed AUNA1 mechanism. This regulatory variant has not been previously reported and reinforces the role of *DIAPH3* in ASND. It also expands the mutational spectrum associated with autosomal dominant auditory neuropathy, highlighting the critical importance of investigating noncoding regions in patients with previously unexplained hereditary HL.

## Methods

### Family Recruitment and Clinical Assessment

The present study received approval from the School of Medicine at Universidade de São Paulo's Ethics Committee and the Brazilian National Committee on Ethics in Research (CONEP; project 67198523.1.0000.0068, approval 6.122.961, June 16, 2023). Written informed consent was obtained from all participants or their legal guardians.

We recruited a large group of consanguineous family members from Pé da Serra do Bilau, an isolated rural community in Sergipe, Brazil (∼ 103 inhabitants). 19 The pedigree showed segregation of HL across 3 to 4 generations, affecting both sexes equally, consistent with autosomal dominant inheritance, though X-linked and autosomal recessive patterns (due to high consanguinity) were also considered.

Seventy-one family members agreed to participate in this study, from whom DNA samples were collected. All participants underwent physical examination and detailed clinical history to exclude syndromic features. Audiological evaluations were conducted in 61 family members, of whom 39 complained of HL, and generally included: pure-tone audiometry (air: 250–8,000 Hz; bone: 500–4,000 Hz); click and bone-conduction ABRs; and distortion-product otoacoustic emissions (DPOAEs). Among the 39 affected individuals, 33 underwent high-resolution temporal bone CT, contributing to a total of 47 family members evaluated for inner ear anatomy ( Supplementary Table S1 ).

### Genetic Testing

#### DNA Extraction and Preliminary Screening

Genomic DNA was extracted from peripheral blood or buccal swabs using commercial kits or a standard salting-out protocol. The affected individuals were prescreened for common pathogenic variants: *GJB2* c.35delG, *GJB6* deletions (del[ *GJB6* -D13S1830] and del[ *GJB6* -D13S1854]), and MT-RNR1 m.1555A > G. Additionally, the *GJB2* coding region was sequenced for at least one individual per sibship.

#### Whole-Exome Sequencing (WES)

Whole-Exome Sequencing was performed on 4 affected individuals (V:8, IV:20, V:10, and IV:3): V:10 and IV:3: Libraries were prepared using the xGen Exome Research Panel (Integrated DNA Technologies) and sequenced on an Illumina HiSeq 2500 (Illumina, Inc.); V:8 and IV:20: Libraries were prepared using the Illumina Nextera Exome Capture system (Illumina, Inc.), and sequenced on a Illumina HiSeq 2500 (Illumina, Inc.).

#### Bioinformatic Processing and Variant Discovery

Initial analysis and filtration primary alignment was performed against the human reference genome (GRCh37/b37) using Burrows-Wheeler Aligner - Maximal Exact Matches (BWA-MEM Wellcome Trust Sanger Institute), followed by variant calling according to GATK Best Practices. Initial annotation utilized ANNOVAR (QIAGEN Digital Insights) and SnpEff (open source), with frequency filtering against the 1000 Genomes Project (1kGP), Exome Aggregation Consortium (ExAC), and Arquivo Brasileiro Online de Mutações (ABraOM) databases. During the primary analysis conducted approximately a decade ago, variant prioritization was performed via Variant Annotation, Analysis, and Search Tool (VAAST) (Fabric Genomics) and Polymorphism Phenotyping v2 (PolyPhen-2) (Harvard Medical School), integrating functional impact predictions with family segregation data.

### Structural Variant (SV) Detection and AI-Driven Re-analysis

To address the unexplained genetic basis of the phenotype within the pedigree, we recently re-evaluated the WES data using the Emedgene AI platform (Illumina). This platform facilitated integrated alignment, calling, and manual curation of single-nucleotide variants (SNVs), small indels, and structural variants (SVs).

To ensure robust detection of non-coding structural changes, we employed a multi-caller consensus strategy, as follows: breakpoint evidence – Manta was used to identify SVs supported by split-read and paired-end evidence; coverage analysis – ExomeDepth (v1.1.10), CNVkit, XHMM, and panelcn.MOPS were utilized to detect copy-number variations (CNVs); consensus & annotation – SV candidates were annotated using AnnotSV, Veronique Geoffroy, to generate a consensus Variant Call Format.files (VCF).

Final prioritization and manual Binary Alignment Map (BAM) inspection focused on rare, non-coding regulatory variants in the 5' untranslated region (5' UTR) of known HL genes that were poorly captured or missed by previous pipelines.

### Validation of the *DIAPH3* 5' UTR Deletion

A heterozygous *DIAPH3* 5'UTR deletion was identified via split-read analysis and found to be absent from the gnomAD database (v2, v3, and v4 SV callsets), supporting its rarity. The variant was absent from public population databases, structural variant catalogs, and from a large internal dataset of Brazilian exomes and genomes, 20 supporting its rarity and strengthening its candidacy as the causal lesion (gnomAD/ Genome, gnomAD/ Exome, Deafness Variation Database, Database of Genomic Variants [DGV], Clinical Genome Resource [ClinGen] copy-number variants [CNVs]).

The variant was validated using qPCR 21 (PowerUp SYBR Green [Thermo Fisher Scientific]) (2^–ΔΔ deltadelta Ct method) and precisely mapped via 3-primer junction PCR. To assess clinical prevalence, a screening of 100 unrelated individuals with postlingual HL was conducted; no additional carriers were identified, further suggesting the variant is pedigree-specific.

### Sanger Sequencing and Junction Analysis

Breakpoint-spanning amplicons were generated via nested PCR for affected individuals. Products were purified with Exo-SAP (Cellco Biotec) and sequenced on an ABI PRISM 3500 Genetic Analyzer (Applied Biosystems). Chromatograms were aligned to GRCh38/hg38, confirming the deletion boundaries and identifying a small micro-insertion at the repair junction.

### In Silico Analysis

Genomic sequence encompassing the promoter region of the *DIAPH3* was extracted from the human reference genome (GRCh38) within a ± 600 bin window centered on the dominant wild type (WT) cap analysis of gene expression (CAGE) peak (defined as bin 0). Three sequence configurations were analyzed in identical genomic context: WT, patient-specific deletion (DEL), and deletion plus 5 bp insertion (AAGAA; DEL_INS). Predicted transcription initiation activity was generated using AlphaGenome sequence-to-signal modeling for neuronal and blood lineage contexts. Cap analysis of gene expression signal was obtained at single-bin resolution for each construct under identical model parameters.

Differential regulatory activity was quantified by calculating ΔCAGE values (CAGE_variant - CAGE_WT) across all bins for DEL and DEL_INS in both tissues. Positive values indicate predicted gain of transcriptional initiation relative to WT, while negative values indicate loss. Spatially localized peaks were identified by comparing variant and WT profiles across the window using consistent scaling.

To identify nucleotide-specific drivers of regulatory change, in silico saturation mutagenesis was performed across the five inserted bases (positions P0–P4). Each position was systematically substituted with all four nucleotides, and the predicted CAGE signal was recalculated in neuronal and blood contexts. Net regulatory gain relative to WT was computed for each substitution and summarized in 4 × 5 delta matrices, which were visualized as heatmaps to assess positional sensitivity and tissue specificity. All analyses were conducted using consistent computational parameters to ensure comparability across sequence configurations.

## Results

### Clinical Characterization

Of the 71 family members enrolled, DNA was collected and clinical histories obtained for all. Audiological evaluations were completed for 61 subjects; the 10 untested individuals were clinically asymptomatic for HL at the time of the study.

**Fig. 1:**
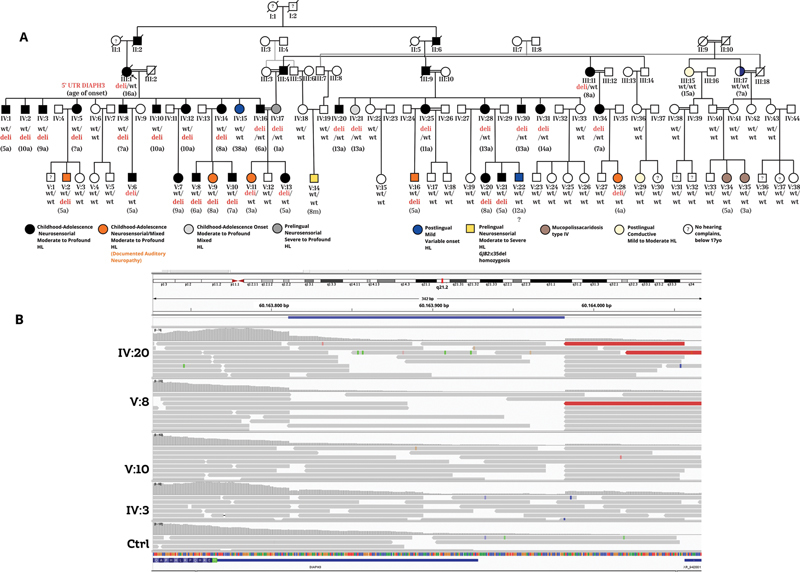
( **A** ) Multigenerational pedigree and segregation analysis. Pedigree of the Brazilian family showing autosomal dominant inheritance of progressive hearing loss. Affected status, reported age at onset (in brackets), and genotype are indicated. ( **B** ) Computational detection via whole exome sequencing (WES). Integrative Genomics Viewer (IGV) snapshot of the *DIAPH3* locus on chromosome 13 across 4 affected family members and control (Ctrl) samples. The data reveal a 171-bp deletion at chr13:60,163,811 (GRCh38), evidenced by a drop in coverage and the presence of split reads and soft-clipped alignments at the breakpoints (delins: heterozygous deletion-insertion; wt: wild type).

A total of 39 individuals from this large multigenerational family from Northeastern Brazil were evaluated for HL ( Fig. 1A ). Eight individuals (III:15, III:17, IV:15, V:14, V:22, V:29, V:34, V:35) were classified as phenocopies based on discordant clinical or molecular findings: 1 individual presented with bilateral prelingual profound HL caused by *GJB2* (OMIM* 121011) c.35del (V:14); 2 sisters were diagnosed with mucopolysaccharidosis type IV (V:34, V:35); 2 individuals had bilateral conductive HL (III:15, V:29); and 3 exhibited subclinical mild HL (III:17, IV:15, V:22).

The remaining 31 affected individuals presented with bilateral, progressive HL with onset between early childhood and adolescence (1–16 years), showing marked intrafamilial phenotypic variability ( Fig. 1A ; Supplementary Table S1 ). Hearing loss was predominantly sensorineural, although a mixed component was identified in three individuals. Five of the 31 affected individuals, demonstrated preserved DPOAEs with absent ABR in at least 1 ear, a pattern consistent with ANSD (V:2, V:9, V:11, V:16, V:28). Temporal bone imaging did not reveal cochlear or vestibular malformations in the individuals evaluated.

### Genomic Analysis

Multiple rounds of reanalysis of WES, performed over 10 years, in 4 affected individuals failed to identify plausible coding single-nucleotide variants or small insertions/deletions segregating with the phenotype, despite clear evidence of an autosomal dominant inheritance pattern observed in the pedigree ( Fig. 1A , Supplementary Table S1 ). Earlier analyses relied primarily on depth-based CNV detection tools, such as ExomeDepth, which have limited sensitivity for small insertions and deletions (< 1–2 kb), typically classified as SVs, particularly in the context of uneven exome coverage.

**Fig. 2:**
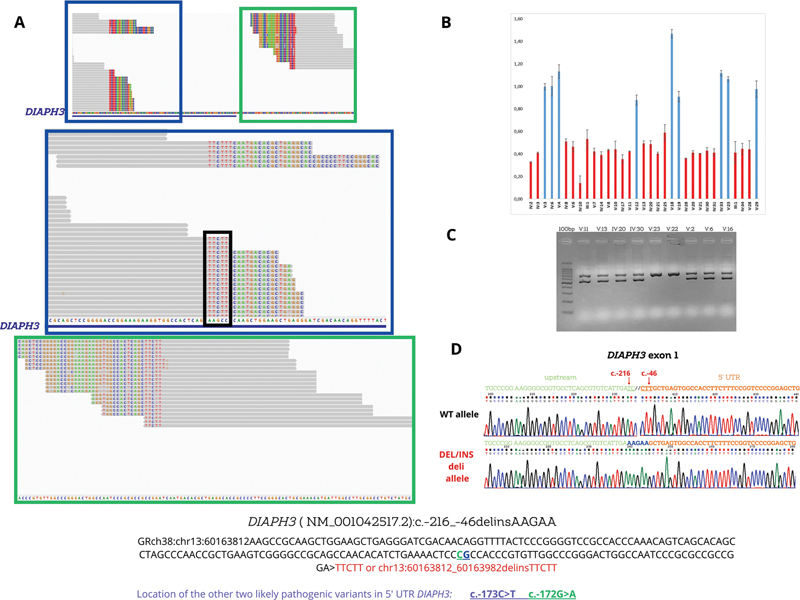
( **A** ) High-resolution breakpoint analysis. Top panel: Broad view of the structural variant in IGV. Middle panel: Magnification of the left portion of the image above. The black box indicates the junctional AAGAA sequence, consistent with a small insertion accompanying the deletion event. Bottom panel: Magnification of the right portion of the image above. (B) Copy number validation by qPCR. Representative quantitative polymerase chain reaction (PCR) results for *DIAPH3* exon 1. An exon-2 amplicon was used as an internal reference for normalization, confirming the loss of 1 copy of the 5'UTR region in affected individuals. ( **C** ) PCR-based segregation. Representative 2% agarose gel electrophoresis of the *DIAPH3* 5'UTR. Carriers of the variant exhibit a doublet consisting of the wild-type band and a shorter band (∼ 170 bp less), consistent with the deletion. ( **D** ) Refined breakpoint confirmation by Sanger sequencing. Chromatograms of the *DIAPH3* 5'UTR. The upper panel shows the wild-type sequence. The lower panel, obtained after nested PCR and sequencing of the gel-excised mutant band, identifies the exact deletion coordinates and the five-nucleotide insertion (AAGAA) at the junction.

A comprehensive reanalysis of archival WES data using updated split-read and paired-end structural-variant detection algorithms identified a previously undetected structural alteration in the 5' UTR of *DIAPH3* ( Fig. 1B ). Visual inspection of BAM files and analysis with the structural variant (SV) caller Manta confirmed a discrete deletion with an complex insertion (indel) spanning the *DIAPH3* promoter region characterizing a strucutural variant ( Fig. 2A ).

Quantitative PCR validation was performed on 56/61 individuals; the remaining 5 samples were excluded due to low-integrity DNA. Despite these exclusions, the variant's co-segregation with the progressive sensorineural hearing loss (SNHL) phenotype was clearly established ( Fig. 2B ).

A breakpoint-specific PCR assay was subsequently designed to amplify the region spanning the deletion, yielding a smaller amplicon corresponding to the SV allele ( Fig. 2C ). This assay confirmed the presence of the structural variant (SV) in all 31 affected individuals (black symbols in Fig. 1A ) and its absence in 40 additional family members, including all phenocopies. The same assay was used to screen 100 unrelated individuals with postlingual progressive HL of unknown genetic etiology evaluated in our laboratory, and no additional carriers were identified. The variant was absent from population databases and structural variant repositories.

To precisely characterize the breakpoint, the PCR product corresponding to the SV allele was gel-extracted, purified, and used as a template for a second round of PCR. The resulting nested PCR product was subjected to Sanger sequencing ( Fig. 2D ). This analysis revealed a 170-bp deletion with a 5-bp insertion, consistent with a complex indel, annotated as *DIAPH3* : c.–216_–46delinsAAGAA. Segregation analysis using both qPCR and breakpoint-specific PCR demonstrated complete co-segregation of the variant with the word characteristic HL phenotype, with no unaffected carriers or affected non-carriers identified in the family.

### Functional Modeling of Regulatory Remodeling

To investigate the regulatory consequences of the patient-specific deletion (DEL) and subsequent 5-bp (AAGAA) insertion (DEL_INS) at the *DIAPH3* locus, we predicted CAGE signal profiles across a 600-bin window. Given the specialized nature of hair cells as modified neurons, neuronal lineage tracks were utilized as the primary model for sequence-to-expression prediction.

**Fig. 3:**
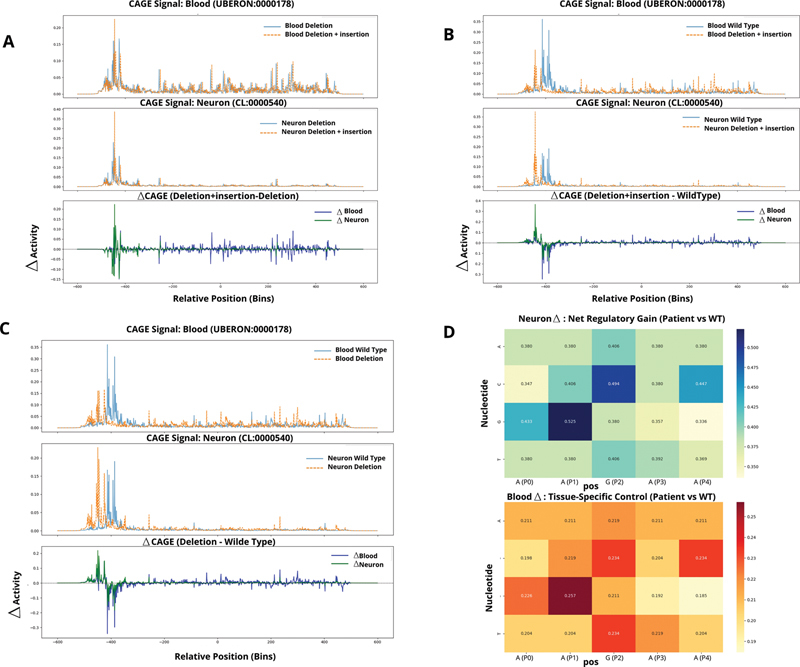
Deep-learning based regulatory profiling and sensitivity mapping of the pathogenic *DIAPH3* locus. Panels **A–C** utilize the AlphaGenome model to map transcription start site (TSS) activity within a 600-bin window, illustrating the spatial distribution of genomic signals across 3 comparative states. Panel A isolates the specific regulatory impact of the 5 bp AAGAA insertion, demonstrating a sharp de novo spike in neuronal activity at position -444. Panel B illustrates the global regulatory transition from a blood-dominant promoter in the wild-type to a neuronal-dominant enhancer state in the patient, while panel C visualizes the regulatory silencing and loss of native blood-specific signals caused by the patient's deletion alone. The corresponding ΔCAGE tracks at the bottom of each panel quantify the net regulatory shift, with green peaks representing a gain in neuronal activity and blue peaks signifying a loss in blood-specific signals. Panel **D** provides high-resolution in silico saturation mutagenesis heatmaps to map the functional sensitivity of the 5 bp insertion relative to the healthy wild-type baseline. The x-axis displays the original patient insertion sequence indexed from P0 through P4, while the y-axis represents the 4 potential nucleotides introduced as trial alleles at each position. The neuron track identifies a high-magnitude gain-of-function core at positions P1 and P2, reaching a maximum Δ of 0.525, characterizing the creation of a potent neuronal enhancer. The blood track serves as a tissue-specific control, showing significantly lower regulatory gain with a maximum Δ of 0.257, confirming the auditory-specific nature of the AUNA1 phenotype.

In the WT configuration, *DIAPH3* exhibits robust promoter-associated activity in the blood lineage, characteristic of its GC-enriched regulatory architecture. In contrast, baseline neuronal activity is comparatively modest. Introduction of the deletion (DEL) markedly attenuates the blood-lineage CAGE signal, indicating a significant disruption of the canonical promoter landscape. Crucially, the subsequent 5-bp insertion does not restore the WT regulatory profile. Instead, the DEL_INS allele induces a pronounced, spatially restricted spike in the neuronal CAGE track centered at relative bin −444, reaching a predicted magnitude of 0.38 CAGE units. This peak is absent in both WT and DEL configurations, demonstrating that the combined variant introduces a de novo regulatory feature rather than rescuing endogenous promoter function ( Figs. 3A–C ).

Quantitative ΔCAGE analysis (mutant versus WT) further elucidates this shift in regulatory logic as follows: neuronal track: The DEL_INS variant generates a sharp positive delta peak at −444, signifying a localized gain of transcriptional potential. Blood lineage: Activity remains suppressed relative to WT, with no compensatory gain at the insertion site ( Fig. 3 ). These data demonstrate that the DEL_INS mutation does not result in a global, non-specific enhancement of *DIAPH3* expression. Instead, it triggers a tissue-selective gain-of-function specifically within the neuronal context. The spatial precision of this CAGE spike suggests the emergence of a discrete, ectopic regulatory module—potentially a de novo transcription factor binding site—rather than diffuse chromatin remodeling.

To identify the nucleotide determinants underlying this neuronal gain, we performed in silico saturation mutagenesis across the 5 inserted bases (A-A-G-A-A; positions P0–P4) and calculated the net regulatory gain relative to the WT baseline. The neuron-specific delta heatmap revealed a high-sensitivity regulatory core centered on positions P1 and P2 ( Fig. 3D ). The maximum positive shifts were observed at P1 (Δ = 0.525) and P2 (Δ = 0.494), with elevated values preferentially associated with guanine-cytosine (GC)-enriched substitutions.

This concentrated sensitivity indicates that specific nucleotide identities at these positions substantially increase predicted neuronal transcriptional activity, consistent with enhanced recruitment or stabilization of GC-responsive transcriptional machinery within neuronal chromatin context. In contrast, the blood-lineage delta heatmap displayed a markedly lower dynamic range, with a maximum Δ of 0.257 and no comparable high-intensity core. The absence of strong positional sensitivity in blood cells indicates that the regulatory motif architecture introduced by the insertion is selectively active in neurons and comparatively inert in systemic tissues.

Collectively, these analyses support a model in which the deletion disrupts the baseline promoter configuration at the *DIAPH3* locus, while the subsequent AAGAA insertion reconfigures local sequence architecture to favor a GC-sensitive regulatory element that selectively enhances neuronal transcriptional potential. The effect is spatially restricted, nucleotide-dependent, and tissue-specific, consistent with a gain-of-function regulatory reprogramming event rather than restoration of canonical promoter activity.

This neuron-restricted regulatory enhancement provides a mechanistic framework for selective vulnerability in AUNA1, in which pathogenic overactivation of *DIAPH3* occurs in auditory neurons and/or inner hair cells despite relatively limited dysregulation in systemic tissues.

## Discussion

In the current study, we identified a novel heterozygous 170-bp deletion/5-bp insertion in the 5'UTR of *DIAPH3* (c.-216_-46delinsAAGAA) segregating with autosomal dominant progressive HL in a large Brazilian pedigree. To our knowledge, this represents the most extensive regulatory *DIAPH3* variant reported to date, significantly expanding the mutational spectrum of AUNA1.

### Clinical Heterogeneity and Disease Evolution

The clinical presentation within this cohort was notably heterogeneous, spanning from “classic” ANSD, characterized by preserved DPOAEs and absent ABRs, to advanced with age, progressive SNHL. This phenotypic breadth reinforces the emerging consensus that *DIAPH3* -related pathology is not strictly limited to an electrophysiological ANSD signature. Instead, the disorder appears to evolve toward broader cochlear dysfunction as the disease progresses.

The pathogenicity of this variant is supported by its complete absence from global population databases (gnomAD), structural variant catalogs (DGV, ClinVar), and a large internal dataset of Brazilian exomes. The perfect co-segregation of this complex indel across 31 affected carriers strongly suggests it is the primary driver of the previously unresolved genetic architecture in this family. By identifying this non-coding SV, we highlight the critical role of large-scale genomic alterations in unresolved cases of hereditary deafness.

### Molecular Mechanisms: From Actin to Synapse

Auditory neuropathy spectrum disorder arises from diverse molecular defects affecting IHCs, ribbon synapses, or spiral ganglion neurons (SGNs). 5 6 7 8 Accordingly, pathogenic variants in genes such as *OTOF, DIAPH3, ATP11A* , and *TMEM43* demonstrate that ANSD represents a convergent electrophysiological phenotype reflecting dysfunction across multiple cochlear compartments ( Table 1 ). Within this framework, *DIAPH3* is a key link between cytoskeletal architecture and synaptic fidelity. It encodes a diaphanous-related formin essential for linear actin nucleation and microtubule stabilization. In the cochlea, *DIAPH3* expression is vital for maintaining stereocilia morphology and ribbon synapse organization. Unlike genes affecting mechanotransduction, *DIAPH3* pathology results from gain-of-function overexpression. 11 14 15 22

**Table 1:** Genes associated with non-syndromic auditory neuropathy/synaptopathy

Gene	Protein	Locus	IP	Proposed mechanism	Evidence (ClinGen)	Auditory phenotype peculiarities	Reference
*OTOF* (MIM*603681) *2p23.2*	Otoferlin	DFNB9/ AUNB1	AR	Presynaptic Ca ^2+^ -sensor for synaptic vesicle exocytosis at IHC ribbon synapse → neurotransmitter release.	Definitive for AR SNHL 9.	Classic cause of congenital ANSD: OAEs often preserved, ABR absent. Many reported pathogenic variants.	36
*PJVK* (MIM*610219) *2q31.2*	Pejvakin	DFNB59	AR	Neuronal/neurite oxidative-stress responses (peroxisome/pexophagy related).	Definitive for SNHL.	Prelingual (often preserved OAEs early).	37
*DIAPH3* (MIM*614567) *13q21.3*	Diaphanous Homolog 3	AUNA1	AD	Actin/cytoskeleton regulation in IHCs; overactivity reduces IHC ribbon synapses → synaptopathy.	Limited for HL.	Typical late-onset progressive auditory neuropathy.	14 40
*SLC17A8* (MIM*607557) *12q23.1*	VGLUT3	DFNA25	AD	Vesicular glutamate transporter in IHCs—required for glutamate loading of synaptic vesicles.	Definitive for SNHL.	Progressive, high-frequency, Affected male members reported an earlier onset and were more severely affected.	41
*CABP2* (MIM*607314) *11q13.2*	Calcium-binding protein 2	DFNB93	AR	Modulator of L-type voltage-gated calcium channels (Ca _v_ 1.3, encoded by *CACNA1D* ).	Definitive for SNHL.	Prelingual U-shaped audiogram; normal static compliance on tympanometry, and middle ear pressures on tympanometry.	42
*ROR1* (MIM*602336) *1p31.2*	Receptor tyrosine kinase like orphan receptor 1	DFNB108	AR	Possible role in hair-cell differentiation and stereocilia organization.	Limited for SNHL.	Congenital, also associated with Fusion of the cochlea and vestibule into a common cavity.	43
*TMEM43* (MIM*612048) *3p25.1*	Transmembrane protein 43	DFNA100/ AUNA3	AD	Structural and electrical coupling between inner border supporting cells and auditory neurons at the IHC-SGN junction.	Not reviewed.	Progressive hearing loss with inability to discriminate speech but preserved sensitivity to sound.	44
*AIFM1* (MIM*300169) *Xq26.1*	Apoptosis-inducing factor, mitochondria-associated 1	AUNX1	XL	Neuronal vulnerability.	Moderate for Leigh syndrome.	HL, sometimes progressive; isolated auditory phenotype or with additional neurologic features depending on variant.	9 10
*ATP11A* (MIM*605868) *13q34*	ATPase phospholipid transporting 11A - phospholipid flippase	AUNA2/ DFNA84	AD	Maintenance of membrane lipid asymmetry critical for stereocilia stability, hair-cell membrane curvature, synaptic function.	Moderate for AD NS HL.	Postlingual progressive HL, abnormal ABR even before the onset of symptoms; Loss of TEOAE as disease progresses, Absent stapedius reflexes; Age at onset infancy to adult.	45

**Abbreviations** : ABR, auditory brainstem response; IP=Inheritance Pattern, AR=Autosomal Recessive. AD=Autosomal Dominant, NS=non-syndromic; SN= sensorineural; ClinGen, Clinical Genome Resource; HL, hearing loss; IHC, inner hair cells; OAE, otoacoustic emissions; SGN, spiral ganglion neuron; SNHL, sensorineural hearing loss; TEOAE, transient evoked otoacoustic emissions.

The *DIAPH3* gene encodes a diaphanous-related formin involved in linear actin nucleation and elongation, with additional roles in microtubule stabilization. 15 23 24 Its expression in both IHCs and SGNs is essential for maintaining stereocilia morphology, vesicle trafficking, and ribbon synapse organization. 11 25 Unlike classical deafness genes that primarily affect mechanotransduction structures, *DIAPH3* -associated disease appears to result from dysregulated actin polymerization and synaptic disorganization, producing a mechanism that preferentially predisposes to neural and synaptopathic forms of HL. 9 26

The molecular landscape of AUNA1 has traditionally been defined by 5'UTR point mutations (c.-172G > A and c.-173C > T variants) that disrupt a conserved GC-box repressor binding site. 11 14 22 While these substitutions cause subtle regulatory interference, our 170-bp deletion/5-bp-insertion likely removes multiple transcription factor binding sites and secondary mRNA structures. This could result in transcriptional disinhibition, causing an excess of *DIAPH3* that over-stabilizes actin filaments. This “cytoskeletal disruption” would trigger the elongation and fusion of IHC stereocilia, followed by the “synaptic failure” of ribbon synapses. This sequence would explain the hallmark clinical presentation: absent ABRs (neural/synaptic failure) despite preserved OAEs (outer hair cell survival).

### Diagnostic Challenges and Translational Implications

A major contribution of the current study is the identification of a variant in a region poorly captured by conventional exome sequencing. 27 This family remained genetically unresolved for decades due to low sequencing coverage of the *DIAPH3* 5'UTR and the reduced sensitivity of standard CNV tools in untranslated regions. 28 Only through breakpoint-aware SV detection and manual BAM inspection was the causative lesion identified. This highlights the need for systematic inclusion of untranslated regulatory regions and structural variant analysis in the diagnostic workflow of large pedigrees with clear autosomal dominant segregation. 29 30

Tissue-specific differences, as observed in ΔCAGE profiles, further underscore the context-dependent nature of regulatory variation. 31 Neuronal tissues exhibit heightened sensitivity to such perturbations, which may explain the variability of auditory phenotypes among carriers.

From a translational perspective, these findings support the feasibility of rational genome engineering approaches aimed at restoring regulatory output. 33 The partial rescue predicted for the insertion allele suggests that fine-scale sequence design could be optimized to improve promoter performance in a tissue-dependent manner. In this context, deep learning-based frameworks like AlphaGenome represent valuable tools for prioritizing candidate regulatory edits before experimental validation. 31 32

From a clinical and diagnostic standpoint, our findings indicate a shift in how familial HL is managed, particularly when initial exome results are negative. We strongly recommend that diagnostic panels for hereditary deafness be expanded to include targeted sequencing of the *DIAPH3* 5'UTR and its upstream regulatory region, as these non-coding areas are frequently overlooked or poorly captured by standard exome platforms. 34 35 36 Furthermore, the implementation of mandatory structural variant (SV) calling using breakpoint-aware algorithms, such as Manta or Lumpy, should be standard practice for all unresolved dominant cases to identify large-scale, non-coding alterations. 34 35 36 Finally, clinicians should adopt a strategy of long-term audiological monitoring that recognizes the potential for AUNA1 to evolve from a “pure” ANSD pattern into SNHL. Serial evaluations involving both DPOAEs and speech perception testing are essential to track this progression and to precisely guide the timing for interventions such as cochlear implantation. 37 38 39

## Conclusion

While our predictive models suggest complex regulatory remodeling—potentially involving shifts in transcription start sites—functional validation remains necessary to fully capture higher-order chromatin interactions. Furthermore, the elevated frequency of DIAPH3-HL in this geographically isolated settlement likely reflects a founder effect.
