# Supplementary material for: A Structural Variant in the 5' Regulatory Region of DIAPH3 Segregates with Postlingual Hearing Loss and Auditory Neuropathy in a Multigenerational Brazilian Family
Source: Int Arch Otorhinolaryngol. 2026 May 5;30(2):1–11. doi: 10.1055/a-2832-6312 (PMC13288208; doi:10.1055/a-2832-6312)
Supplement: Supplementary file 1 — Supplementary Material [file 10-1055-a-2832-6312-s262265.pdf]

Table S1: Clinical and genetic summary. Legend: BNSHL-PP=bilateral neurosensorial hearing loss - postlingual and progressive; NS=neurosensorial; cond.=conductive; A= absent; P= present; nl=normal, NLH=normal-heanring; prof.=profound; sev-prof.=severe-profound; mod.=moderate; nl=normal

Classification	pedigree position	Gender	age of onset	Type	Laterality	age at audio	degree	OEAPD RE	OEAPD LE	BERA	5'UTR *DIAPH3* DELETION all	WES	qPCR	BK PCR
BNSHL-PP	**III:1**	F	16	NS	Bi	65	prof.	A	A	A	**P**	**P**	**P**	**P**
BNSHL-PP	**III:11**	F	8	NS	Bi	ND	prof.	A	A	N.A.	**P**	N.T.	**P**	**P**
BNSHL-PP	**IV:1**	M	5	NS	Bi	21	prof.	A	A	A	**P**	N.T.	**P**	**P**
BNSHL-PP	**IV:2**	M	10	NS	Bi	26	prof.	A	A	A	**P**	N.T.	**P**	**P**
BNSHL-PP	**IV:3**	M	9	NS	Bi	29	prof.	A	A	A	**P**	**P**	**P**	**P**
BNSHL-PP	**IV:5**	F	?	NS	Bi	31	prof.	A	A	A	**P**	N.T.	**P**	**P**
BNSHL-PP	**IV:8**	M	?	NS	Bi	35	sev-prof.	A	A	A	**P**	N.T.	**P**	**P**
BNSHL-PP	**IV:10**	M	10	NS	Bi	35	prof.	A	A	A	**P**	N.T.	**P**	**P**
BNSHL-PP	**IV:12**	F	10	NS	Bi	36	prof.	A	A	A	**P**	N.T.	**P**	**P**
BNSHL-PP	**IV:14**	F	8	NS	Bi	41	prof.	A	A	A	**P**	N.T.	**P**	**P**
BNSHL-PP	**IV:16**	M	6	NS	Bi	42	prof.	A	A	A	**P**	N.T.	**P**	**P**
BNSHL-PP	**IV:17**	F	1	NS	Bi	16	prof.	A	A	A	**P**	N.T.	**P**	**P**
BNSHL-PP	**IV:20**	M	13	NS	Bi	28	sev-prof.	A	A	A	**P**	**P**	**P**	**P**
BNSHL-PP	**IV:21**	F	13	mixed	Bi	29	sev-prof.	A	A	A	**P**	N.T.	**P**	**P**
BNSHL-PP	**IV:25**	F	11	NS	Bi	33	sev-prof.	A	A	A	**P**	N.T.	**P**	**P**
BNSHL-PP	**IV:28**	F	13	NS	Bi	34	prof.	A	A	A	**P**	N.T.	**P**	**P**
BNSHL-PP	**IV:30**	M	13	NS	Bi	35	prof.	A	A	A	**P**	N.T.	**P**	**P**
BNSHL-PP	**IV:31**	F	14	NS	Bi	37	prof.	A	A	A	**P**	N.T.	**P**	**P**
BNSHL-PP	**IV:34**	F	7	NS	Bi	29	prof.	A	A	A	**P**	N.T.	**P**	**P**
BNSHL-PP	**V:2**	M	5	mixed	Bi	10	mod.-sev.	**P**	**P**	A	**P**	N.T.	**P**	**P**
BNSHL-PP	**V:6**	M	5	NS	Bi	8	prof.	N.A.	N.A.	N.A.	**P**	N.T.	**P**	**P**
BNSHL-PP	**V:7**	F	9	NS	Bi	10	sev-prof.	A	A	A	**P**	N.T.	**P**	**P**
BNSHL-PP	**V:8**	M	6	NS	Bi	15	prof.	A	A	A	**P**	**P**	**P**	**P**
BNSHL-PP	**V:9**	F	8	NS	Bi	21	prof.	**P**	A	A	**P**	N.T.	**P**	**P**
BNSHL-PP	**V:10**	M	7	NS	Bi	21	prof.	A	A	A	**P**	**P**	**P**	**P**
BNSHL-PP	**V:11**	F	3	NS	Bi	12	prof.	**P**	**P**	A	**P**	N.T.	**P**	**P**
BNSHL-PP	**V:13**	F	5	NS	Bi	16	sev-prof.	A	A	A	**P**	N.T.	**P**	**P**
BNSHL-PP	**V:16**	M	5	NS	Bi	6	mod.	**P**	**P**	N.A.	**P**	N.T.	**P**	**P**
BNSHL-PP	**V:20**	F	8	N.A.	N.A.	ND	N.A.	N.A.	N.A.	N.A.	**P**	N.T.	**P**	**P**
BNSHL-PP	**V:21**	M	5	NS	Bi	5	mild-mod.	A	A	A	**P**	N.T.	**P**	**P**
BNSHL-PP	**V:28**	F	4	mixed	Bi	7	sev-prof.	**P**	**P**	A	**P**	N.T.	**P**	**P**
Cond. HL	**III:15**	F	15	cond.	Bi	80	mod.	A	A	A	A	N.T.	N.T.	A
mild HL	**III:17**	F	?	NS	Uni	78	mild	P	P	nl	A	N.T.	N.T.	A
BNSHL-PRE	**V:14**	M	<1	NS	Bi	9	mod-sev.	A	A	A	A	N.T.	N.T.	A
mild HL	**IV:15**	F	38	NS	Bi	38	mild-mod.	A	A	nl	A	N.T.	A	A
mild HL	**V:22**	M	12?	NS	Bi	12	mild	P	P	nl	A	N.T.	A	A
Cond. HL	**V:29**	F	-	cond.	Bi	17	N.A.	A	A	nl	A	N.T.	A	A
Morquio S.	**V:34**	F	5	NS	Bi	20	mod-sev.	A	A	A	A	N.T.	N.T.	A
Morquio S.	**V:35**	F	3	NS	Bi	24	sev-prof.	A	A	A	A	N.T.	N.T.	A
NL-H	**IV:6**	F	-	N.A.	N.A.	32	nl	P	P	nl	A	N.T.	A	A
NL-H	**IV:18**	F	-	N.A.	N.A.	32	nl	P	P	nl	A	N.T.	N.T.	A
NL-H	**IV:19**	M	-	N.A.	N.A.	39	nl	P	P	nl	A	N.T.	N.T.	A
NL-H	**IV:22**	F	-	N.A.	N.A.	31	nl	P	P	nl	A	N.T.	A	A
NL-H	**IV:33**	F	-	N.A.	N.A.	39	nl	P	P	nl	A	N.T.	A	A
NL-H	**IV:36**	F	-	N.A.	N.A.	30	nl	P	P	nl	A	N.T.	A	A
NL-H	**IV:38**	F	-	N.A.	N.A.	33	nl	P	P	nl	A	N.T.	N.T.	A
NL-H	**IV:40**	M	-	N.A.	N.A.	50	nl	P	P	nl	A	N.T.	N.T.	A
NL-H	**IV:41**	F	-	N.A.	N.A.	46	nl	P	A	nl	A	N.T.	N.T.	A
NL-H	**IV:42**	F		N.A.	N.A.	ND	nl	P	P	nl	A	N.T.	N.T.	A
NL-H	**IV:43**	F		N.A.	N.A.	17	nl	P	P	nl	A	N.T.	N.T.	A
NL-H	**V:1**	M	-	-	-	4	N.A.	N.A.	N.A.	N.A.	A	N.T.	A	A
NL-H	**V:3**	F	-	N.A.	N.A.	11	N.A.	N.A.	N.A.	N.A.	A	N.T.	A	A
NL-H	**V:4**	F	-	-	-	2	nl	P	P	nl	A	N.T.	A	A
NL-H	**V:5**	M	-	N.A.	N.A.	8	nl	P	P	nl	A	N.T.	A	A
NL-H	**V:12**	M	-	N.A.	N.A.	14	nl	P	P	nl	A	N.T.	A	A
NL-H	**V:15**	F	-	N.A.	N.A.	10	nl	P	P	nl	A	N.T.	A	A
NL-H	**V:17**	M	-	N.A.	N.A.	11	nl	P	P	nl	A	N.T.	A	A
NL-H	**V:18**	M	-	N.A.	N.A.	13	nl	P	A	nl	A	N.T.	A	A
NL-H	**V:19**	F	-	N.A.	N.A.	12	N.A.	N.A.	N.A.	N.A.	A	N.T.	A	A
NL-H	**V:23**	M	-	N.A.	N.A.	14	nl	P	P	nl	A	N.T.	A	A
NL-H	**V:24**	F	-	N.A.	N.A.	16	nl	P	P	nl	A	N.T.	A	A
NL-H	**V:25**	F	-	N.A.	N.A.	18	nl	P	P	nl	A	N.T.	A	A
NL-H	**V:26**	F	-	N.A.	N.A.	-	N.A.	N.A.	N.A.	N.A.	A	N.T.	A	A
NL-H	**V:27**	M	-	N.A.	N.A.	ND	N.A.	P	P	nl	A	N.T.	A	A
NL-H	**V:30**	F	-	N.A.	N.A.	11	nl.	P	P	nl	A	N.T.	A	A
NL-H	**V:31**	M	-	N.A.	N.A.	19	N.A.	N.A.	N.A.	N.A.	A	N.T.	N.T.	A
NL-H	**V:32**	M	-	N.A.	N.A.	5	nl	A	P	nl	A	N.T.	N.T.	A
NL-H	**V:33**	M	-	N.A.	N.A.	-	N.A.	N.A.	N.A.	N.A.	A	N.T.	A	A
NL-H	**V:36**	M	-	N.A.	N.A.	-	N.A.	N.A.	N.A.	N.A.	A	N.T.	A	A
NL-H	**V:37**	F	-	N.A.	N.A.	-	N.A.	N.A.	N.A.	N.A.	A	N.T.	A	A
NL-H	**V:38**	F	-	N.A.	N.A.	11	nl	P	P	nl	A	N.T.	N.T.	A
